# Editorial note

**DOI:** 10.1098/rsta.2024.0526

**Published:** 2025-05-01

This issue is the second of a two-part issue: ‘Multi-messenger gravitational lensing’. Part 1 can be found at https://royalsocietypublishing.org/toc/rsta/2025/383/2294. The Preface to Part 1 contains an overview of both issues [1], and Smith *et al*. in this issue [2] provide a more extensive introduction to the topic.
